# Early Management of Severe Biliary Infection in the Era of the Tokyo Guidelines

**DOI:** 10.3390/jcm12144711

**Published:** 2023-07-16

**Authors:** Esther Nve, Josep M. Badia, Mireia Amillo-Zaragüeta, Montserrat Juvany, Mónica Mourelo-Fariña, Rosa Jorba

**Affiliations:** 1 Department of Surgery, Hospital Universitari Mútua de Terrassa, 08221 Barcelona, Spain; esthernve@gmail.com; 2 School of Medicine, Universitat Rovira i Virgili, 43003 Tarragona, Spain; rjorba.hj23.ics@gencat.cat; 3 Department of Surgery, Hospital General Granollers, School of Medicine, Universitat Internacional de Catalunya, Av Francesc Ribas 1, 08402 Granollers, Spain; mamillo@fphag.org (M.A.-Z.); mjuvany@fphag.org (M.J.); 4 Intensive Care Unit, Complexo Hospitalario Universitario A Coruña, 15006 A Coruña, Spain; monicamourelo@gmail.com; 5 Department of Surgery, Hospital Universitari de Tarragona Joan XXIII, 43005 Tarragona, Spain

**Keywords:** biliary infection, acute cholecystitis, acute cholangitis, acalculous cholecystitis, antibiotic treatment, gallbladder, surgery, review

## Abstract

Sepsis of biliary origin is increasing worldwide and has become one of the leading causes of emergency department admissions. The presence of multi-resistant bacteria (MRB) is increasing, and mortality rates may reach 20%. This review focuses on the changes induced by the Tokyo guidelines and new concepts related to the early treatment of severe biliary disease. If cholecystitis or cholangitis is suspected, ultrasound is the imaging test of choice. Appropriate empirical antibiotic treatment should be initiated promptly, and selection should be performed while bearing in mind the severity and risk factors for MRB. In acute cholecystitis, laparoscopic cholecystectomy is the main therapeutic intervention. In patients not suitable for surgery, percutaneous cholecystostomy is a valid alternative for controlling the infection. Treatment of severe acute cholangitis is based on endoscopic or transhepatic bile duct drainage and antibiotic therapy. Endoscopic ultrasound and other new endoscopic techniques have been added to the arsenal as novel alternatives in high-risk patients. However, biliary infections remain serious conditions that can lead to sepsis and death. The introduction of internationally accepted guidelines, based on clinical presentation, laboratory tests, and imaging, provides a framework for their rapid diagnosis and treatment. Prompt assessment of patient severity, timely initiation of antimicrobials, and early control of the source of infection are essential to reduce morbidity and mortality rates.

## 1. Introduction

Sepsis is the leading cause of death worldwide and the most expensive inpatient condition in the United States [1]. Biliary infections are among the main causes of sepsis and emergency department admissions, especially in elderly patients with comorbidities [2]. Its prevalence has increased worldwide due to the increase in invasive diagnostic and therapeutic procedures in the bile duct (via percutaneous, endoscopic, or surgical access) and the increase in complex hepatobiliary surgery, including liver transplantation [3].

The mortality rate of severe biliary infections ranges from 1% to 6% [4]. However, when combined with bacteremia (which occurs in 10% of cholecystitis and 50% of cholangitis cases), mortality can rise to 10–20% [5,6]. Bacteraemia of the biliary tract accounts for up to 20% of community bacteraemia in the elderly community [7] and is the second most frequent cause of sepsis in this age segment [8,9].

The first edition of the Tokyo Guidelines (TG07) [10] made significant advances in consolidating definitions and establishing the diagnostic and severity criteria for biliary tract infections. Its three successive editions were based on an extensive review of the evidence, the development of new diagnostic and treatment strategies, and the performance of studies to validate them.

Although the TGs have undoubtedly contributed to homogenizing the approach to treating these frequent and very serious infections, they have come under some criticism in the West [11,12,13], especially with regard to certain details of the treatment algorithms. A survey on adherence to the TGs conducted in 25 hospitals in nine Western countries showed a low level of compliance, especially in initial surgical management, with the guidelines being applied in around 50% of cholecystectomies during the first admission and in culture-directed antibiotic therapy, where they were applied in only 25% of the cultures performed [13].

The TGs classify biliary infections into mild, moderate, and severe and rely on local and systemic signs of inflammation, imaging findings, and an organ failure score to determine the grade of severity and the management alternatives. Some authors have analyzed the impact of the application of the TGs on outcomes, but no substantial benefits have been found, for instance, in the treatment of cholecystitis [14]. These findings have been integrated into the 2013 (TG13) [15] and 2018 (TG18) [16] editions, with some modifications to the severity criteria and the recommendations for antimicrobial treatment and source control.

The aim of this study was to conduct a systematic review of recommendations for the management of severe biliary infection following the new definitions of sepsis, the dissemination of the TGs and the development of new minimally invasive endoscopic techniques.

## 2. Materials and Methods

A review of the medical literature was carried out in accordance with the recommendations of the Cochrane Handbook [17] and the PRISMA reporting method [18]. The literature was searched through PubMed and The Cochrane Library. The search was limited to English and Spanish languages and to peer-reviewed articles published from January 1980 to May 2022. MeSH terminology was used for the bibliography research under the topics: acute cholecystitis, acute cholangitis, biliary tract infection, cholecystectomy, cholecystostomy, antibiotic treatment, biliary drainage, antibacterial agents, outcome assessment, mortality, morbidity, survival analysis, cure rate, and treatment success rate.

Preferred inclusion criteria were: controlled clinical studies, cohort studies, clinical practice guidelines, meta-analyses, and systematic reviews. These studies were compiled by two researchers. The final selection of articles and decisions regarding inclusion were made by all researchers jointly.

## 3. Results

**Figure 1:**
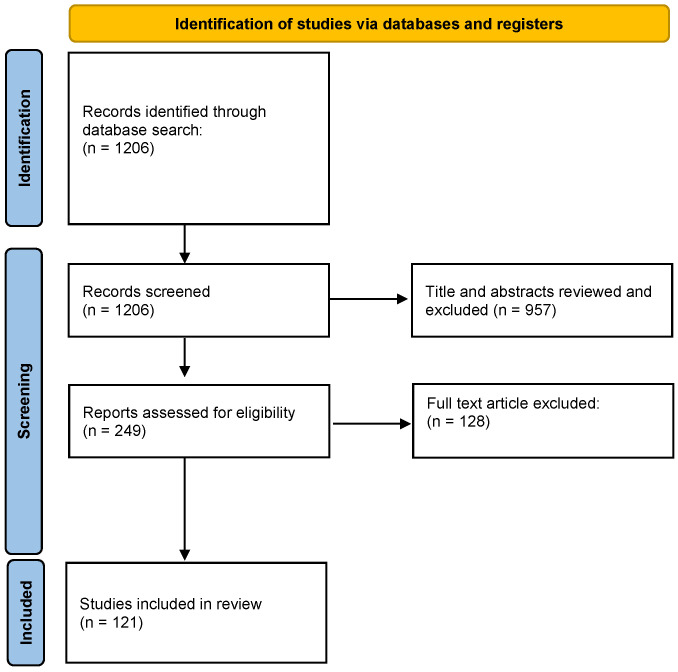
PRISMA flow diagram. From [18].

In total, 1206 publications were identified using the strategy described. After screening by title/abstract, 249 were selected for full-text screening. Of these, 121 were selected for inclusion (Figure 1).

### 3.1. Early Diagnosis

#### 3.1.1. Acute Calculous Cholecystitis

The clinical diagnosis is based on a composite of three variables: sustained pain for more than 12 h in the right upper quadrant, tenderness (with or without Murphy’s sign), and evidence of an acute inflammatory response [19]. However, the classical clinical signs are not very reliable: for instance, Murphy’s sign has a sensitivity of 20% and a specificity of 87% [20]. Acute cholecystitis is categorized in TG18 as severe (with organ dysfunction), moderate (with marked local or systemic inflammatory signs), and mild (no organ dysfunction and minimal inflammatory signs). Table 1 shows the TG18 diagnostic criteria and severity classification of acute cholecystitis [19].

In most cases of acute cholecystitis, a basic set of tests (CBC, liver function, serum amylase, chest X-ray, and abdominal ultrasound) are all that are needed for diagnosis. Mild jaundice (<3.5 mg/dL or 60 µmol/L) may be seen in 20% of patients, owing to inflammation surrounding the bile duct or direct compression by a distended gallbladder [21]. When higher bilirubin levels are observed, choledocholithiasis, cholangitis, or Mirizzi syndrome should be considered. In cholecystitis, the incidence of bile duct stones is not higher than in elective cholecystectomy (3–15%), so minor jaundice does not warrant a preoperative endoscopic retrograde cholangio-pancreatography (ERCP).

Because of its availability and diagnostic efficacy, ultrasound is the initial imaging test of choice in the study of right upper quadrant abdominal pain, even though a 2012 meta-analysis raised doubts about its efficacy, recording a sensitivity of 81% (95% CI: 75–87%) [22]. The most sensitive ultrasound combination is the presence of gallstones and the sonographic Murphy’s sign. Gallbladder wall thickening (>3 mm), as well as pericholecystic fluid, are viewed as secondary findings [23].

#### 3.1.2. Acute Cholangitis

Acute cholangitis requires a combination of biliary obstruction and bacterobilia, the presence of bacterial proliferation in the bile. It presents various levels of severity, ranging from mild cases that improve rapidly only with antimicrobials to more severe cases which have been termed toxic or suppurative cholangitis. Acute bacterial cholangitis is accompanied by shock and mortality rates of 15% and 9%, respectively [6].

It should be noted that the classic triad described by Charcot, comprising jaundice, fever, and right upper quadrant pain, is seen in only 20–50% of patients. It has high specificity but a sensitivity between 21 and 26% [4]. The most severe patients present the Reynolds pentad, with the addition of shock and mental confusion, two signs of organ failure [24]. The diagnosis of acute cholangitis should be considered in any septic patient with abdominal pain and rapid deterioration of their general condition in the absence of an obvious source of infection [4].

The most common etiology of acute cholangitis is incomplete biliary obstruction, usually caused by stones in the bile duct and accompanied by ascending infection. In contrast to acute cholecystitis, enterococci (present in up to 20% of isolates) and anaerobes (50%) need to be considered among the most prevalent causative agents. The infection is polymicrobial in up to 80% of cases and is accompanied by bacteremia in 25–75% of patients [25].

TG18 retains the diagnostic criteria of TG13, showing a diagnostic ability of 90% even though their specificity has not been assessed [4,26] (Table 2). According to TG18, diagnosis is based on a combination of signs of systemic inflammation, cholestasis, and imaging criteria [4]. The TG18/TG13 severity criteria are good predictors of mortality and may select cases requiring emergency biliary drainage.

In addition to gallstones, other etiologies such as primary sclerosing cholangitis, hepatolithiasis, biliary stent obstructions, complications of percutaneous transhepatic cholangiography (PTC) or ERCP, biliary anastomotic stricture and complications of liver transplantation are currently on the rise. In the presence of these etiologies, a change in the bacteriological profile of this disease has been observed in recent years, with greater prominence of *Enterobacter*, *Enterococcus* spp., *Pseudomonas* spp., and resistant enterobacteriaceae in these types of biliary infection.

For the imaging study of acute cholangitis, ultrasound is highly specific for visualizing dilated ducts or gallstones (rates of 96% and 100%, respectively), but its sensitivity is low (42–63%) [27]. In fact, CT is more effective for defining the cause and level of obstruction. Magnetic resonance cholangiopancreatography (MRCP) obtains high-resolution images of the bile duct and pancreatic ducts, avoids contamination of the ducts by contrast medium, is able to show areas proximal and distal to the obstruction, and provides intraluminal and extraluminal images of the biliary tree without causing the morbidity and mortality associated with more invasive techniques. The sensitivity of MRCP for bile duct stones is greater than 90%, although gallstones smaller than 6 mm may be missed. After ultrasound, MRCP is the technique of choice for the study of jaundice and the selection of patients with an indication for therapeutic ERCP [4]. Endoscopic ultrasound (EUS) shows similar accuracy, sensitivity, and specificity to MRCP and ERCP in the diagnosis of common bile duct stones [28]. Cholangiography (identified by ERCP or percutaneous transhepatic biliary drainage, PTBD) requires the injection of contrast under pressure into the obstructed ducts, which carries a risk of cholangitis and bacteremia. However, it provides an accurate view of the bile ducts and allows either temporary or definitive drainage of the biliary tree.

### 3.2. Early Assessment of Severity

Biliary tract infection is a common cause of bacteremia and sepsis. Sepsis is a “life-threatening organ dysfunction caused by a dysregulated host response to infection” that can be recognized by a variety of signs and symptoms in cases with suspected infection [29]. The SIRS criteria were not deemed sufficient to diagnose this condition [30]; serum lactate [31] or organ failure criteria [32], especially those of the SOFA score (Sequential Organ Failure Assessment) [33], were more accurate. QuickSOFA (qSOFA) was proposed in 2018 as a new bedside score, including three variables: a Glasgow scale score of ≤13, a systolic pressure ≤ 100 mm Hg, and a respiratory rate of ≥22/min [34], and has since been under evaluation. Finally, septic shock is defined as a subset of sepsis with profound circulatory and cellular metabolism abnormalities. It is a “state of acute circulatory failure” [35] which results in maintained hypotension necessitating vasopressors to keep MAP ≥ 65 mmHg and a serum lactate level > 2 mmol/L (18 mg/dL), in spite of resuscitation with adequate volume [29].

The new bedside definitions of sepsis should allow early detection of severe biliary infection, optimize treatment, and improve outcomes. However, several authors have raised doubts about qSOFA for the prompt recognition of sepsis [36,37,38,39], finding high specificity but low sensitivity for organ dysfunction (96.1% and 29.7%, respectively) [39]. In one study, qSOFA failed to detect two-thirds of severe sepsis cases [38]. In addition, other studies report low sensitivity of qSOFA in the out-of-hospital setting and emergency department triage, while SIRS criteria perform somewhat better [36]. It may be that the use of qSOFA is not helpful in detecting the earliest phase of sepsis, precisely when early treatment is most needed. In consequence, the Surviving Sepsis Campaign argues against using qSOFA and recommends SIRS, NEWS, or MEWS as a single screening tool for sepsis [40].

If sepsis criteria are present in biliary infection, the revised Surviving Sepsis Campaign [41] “hour-1 bundle” should be triggered, including measurement of serum lactate and blood cultures, resuscitation with intravenous crystalloids and infusion of empirical antibiotics and, in case of life-threatening hypotension, initiation of vasopressor drugs.

### 3.3. Early Administration of Antibiotics

The biliary tree is normally sterile, but colonization occurs in the presence of gallstones, obstruction, biliary stents, or bile-digestive anastomoses. Most biliary infections (80%) are polymicrobial and severe cases are often associated with bacteremia (Table 3). Enteric microorganisms account for the majority of microbiota and are isolated in 70% of cultures [6,42,43,44]. Gram-positive cocci account for 20% of cultures. Among these, Enterococcus spp. is the second most frequent bacterium (found in up to 34% of cases) [44]. Anaerobes are isolated in up to 40% of cases of cholecystitis, 50% of cases of cholangitis, and 72% of cases of gangrenous cholecystitis [45].

The infecting microbiota has changed in recent decades, and distinctions must be made between the infecting flora of cholecystitis and cholangitis and between community-acquired and healthcare-associated infections (HAIs). In a specific analysis of bacteremia of biliary origin [6], increases in *Enterococcus* spp. and *Pseudomonas aeruginosa* were detected in HAIs, including rises of 22% of *E. coli* and *Klebsiella* spp. resistant to fluoroquinolones. It should be noted that the microbiology of stent-bearing bile ducts is very different, with a large presence of enterococci and non-fermenting Gram-negatives, in particular *Enterococcus faecium* and *Pseudomonas aeruginosa* [46]. The increase in *E. faecium* isolates is a cause for concern because of their intrinsic resistance to the usual antimicrobials. Up to 12% of patients with calculous cholecystitis, 17% with acalculous cholecystitis, and 45% with cholangitis have isolates with this organism [47]. The percentage of extended-spectrum beta-lactamase (ESBL)-producing microorganisms is also on the rise [6]. The growing incidence of multidrug-resistant (MDR) organisms may lead to an increase in treatment failure to the extent that some antimicrobials, such as amoxicillin/clavulanate and certain cephalosporins and fluoroquinolones can no longer be used empirically in many regions of the world [44,48].

The combination of early empirical antimicrobial treatment and timely source control is the cornerstone of the successful treatment of severe biliary tract infection. The choice of drug will depend on whether the infection is of community origin or HAI, its severity, and the risk of the biliary tract harboring MRD organisms (Table 4). The suggested empirical antibiotic treatment for biliary infection is shown in Table 5.

Amoxicillin-clavulanic acid, aminoglycosides, piperacillin-tazobactam, and carbapenems are still active against the usual Gram-negatives. However, quinolones are not recommended in some European countries because of their loss of efficacy against these bacteria (between 30 and 55% in the case of resistant *E. coli*) and their low performance against streptococci and enterococci [49]. Gram-positive bacteria maintain a high sensitivity to beta-lactams, except for *E. faecium*, which is sensitive to glycopeptides, daptomycin, and linezolid. Piperacillin-tazobactam, carbapenems, or ceftriaxone achieve high bile concentrations but may select for vancomycin-resistant enterococci [50]. In cases of HAI bacteremia and in patients who are immunocompromised, transplanted, or previously treated with antibiotics, ESBL-producing *Enterobacteriaceae* or *Pseudomonas* spp. may be present, and initial empirical treatment with a carbapenem [6] should be performed.

The choice of antimicrobial therapy in biliary infections depends on the severity of the case, the possibility of adequate source control, and, to a large extent, local antimicrobial data. The TG18 severity categorization can be used to select antibiotics and determine the best approach to septic source control.

#### 3.3.1. Acute Cholecystitis

In mild-moderate cholecystitis, amoxicillin-clavulanic acid (alone or in combination with an aminoglycoside, depending on local *E. coli* resistance) or a cephalosporin combined with metronidazole may be prescribed. Ertapenem is the drug of choice in case of suspected ESBL-producing Enterobacteriaceae. When sepsis or risk factors for antibiotic failure are present, antimicrobial combinations or monotherapy with piperacillin-tazobactam or carbapenem may be chosen initially [44]. In mild-moderate cholecystitis, antibiotics can be withdrawn within 24 h of the cholecystectomy [23], but the treatment should be continued for 3–4 days if there has been gallbladder necrosis, pericholecystic abscess or biliary peritonitis.

#### 3.3.2. Acute Cholangitis

In non-severe community-acquired forms, the regimen is based on 3rd generation cephalosporins (cefotaxime or ceftriaxone), associated with an anaerobic agent if there is a history of bilio-enteric anastomosis. Coverage for *Enterococcus* spp. and *Pseudomonas* aeruginosa should be considered in severe cases, in cases of nosocomial transmission, or immunosuppressed patients. If sepsis is present in HAIs and cases associated with biliary stents, regimens should include a broad-spectrum cephalosporin (cefepime) or a piperacillin + tazobactam combination, in both cases associated with vancomycin and metronidazole in cases involving bilioenteric anastomoses [49].

Empirical antibiotic treatment, together with initial resuscitative measures, is effective in 74–85% of cases, in which biliary decompression can be delayed by 48–72 h [51]. The TG18 guidelines state that early diagnosis, antibiotic treatment, and bile duct drainage are all essential, regardless of the severity of the condition [4].

TG18 recommends 4–7 days of treatment after successful bile duct drainage, except when the etiology is enterococcal or streptococcal, in which it is extended to two weeks to avoid the risk of endocarditis [44]. However, several European publications reduce the duration to three days post-biliary drainage [49,52,53].

### 3.4. Timely Source Control

#### 3.4.1. Early Cholecystectomy

**Figure 2:**
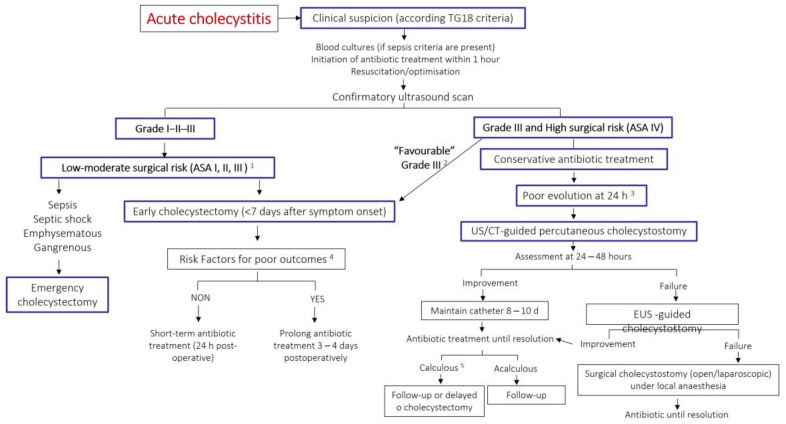
Authors’ proposal for a treatment algorithm for acute cholecystitis. Modified from Okamoto et al. [55]. (1) Patients with acute cholecystitis TG18 Grade I-II-III, suitable for surgery, should be operated on during the first few days of hospitalization. (2) In hospitals with state-of-the-art HPB surgery departments, some Grade III patients, despite being at high risk, may benefit from early advanced resuscitation and become fit for surgery within the first few days of hospital admission. (3) Patients with non-favorable Grade III who do not improve with antibiotic treatment may benefit from an imaging or EUS-guided cholecystostomy. (4) Risk factors for poor outcomes may be surgical (gangrenous gallbladder, perivascular abscess, choleperitoneum) or the general risk factors listed in Table 4. (5) In calculous cholecystitis successfully treated with cholecystostomy, deferred surgical treatment should be considered. If a high anesthetic risk persists, non-surgical treatment of stone disease or clinical follow-up may be considered. TG18: Tokyo guidelines 2018. “Favorable” Grade III: Favorable organ system failure (FOSF), absence of negative predictive factors, good performance status (PS). FOSF: rapidly reversible cardiovascular or renal failure. Negative predictive factors: jaundice, neurological dysfunction, respiratory dysfunction. PS: Charlson > 4. US: ultrasound. EUS: endoscopic ultrasound. CT: computerized tomography. ASA: American Society of Anesthesiologists surgical risk classification.

The clinical outcomes of early cholecystectomy (performed within 7–10 days of symptom onset [16]) are better than those of delayed surgery (antibiotic treatment and surgery after 5–6 weeks). Meta-analyses comparing early and delayed surgery found no differences in morbidity and mortality [22,54] or in the percentage of bile duct injury with either open or laparoscopic techniques. The TG18 algorithms rely on the degree of gallbladder inflammation and severity assessment to decide on cholecystectomy [55]. Similar to many standard protocols, they indicate laparoscopic cholecystectomy in Grade I and II cholecystitis, and even in Grade III, provided there are no negative predictive factors, the patient has a good performance status, favorable organ system failure, and is attended at an advanced center with access to intensive care and advanced laparoscopic techniques [55,56,57]. Figure 2 summarizes a proposed algorithm for the management of acute cholecystitis.

#### 3.4.2. Cholecystostomy

Placement of a percutaneous ultrasound- or CT-directed drainage catheter (PT-GBD) in the gallbladder is an accepted alternative in critically ill patients, those with septic shock, and high-risk subjects for general anesthesia. Its success rate in calculous cholecystitis is 90.7% [58]. Indications for cholecystostomy seem to be more extensive in the TG18 guidelines than in Western practice [59]. No trials comparing emergency cholecystectomy and cholecystostomy in elderly or high-risk patients have been identified; one systematic review in critically ill patients found insufficient evidence to support the TG18 recommendation in favor of percutaneous drainage over emergency surgery [58]. It concluded that “cholecystectomy may be a better alternative to cholecystostomy for acute cholecystitis in the elderly and/or critically ill.” In fact, the mortality of emergency cholecystectomy is between 0 and 1.5% in patients over 65 [58] or 70 years of age [60,61]. Cholecystostomy is the definitive treatment for acalculous cholecystitis, but patients with gallstones should be assessed for cholecystectomy, as the risk of re-admission for biliary causes is 49% in the first year [62]. Patients who are definitely not suitable for surgery may be offered non-surgical treatments for gallstones or stone removal through the cholecystostomy route after six weeks of the acute process.

Recently, endoscopic ultrasound-guided cholecystostomy (EUS-GBD) using an anti-migrating tubular self-expandable or lumen-apposing metal stent has been added to the therapeutic armamentarium to treat acute cholecystitis in patients unsuitable for surgery [63]. A systematic review and meta-analysis comparing EUS-GBD with PT-GBD found that PT-GBD may be associated with a slightly higher risk of periprocedural complications, such as bleeding or injury to surrounding structures [64]. Although both provided effective symptom relief and may serve as temporary measures to stabilize patients until a subsequent elective cholecystectomy, EUS-GBD may have a lower risk for subsequent interventions, potentially reducing the need for additional procedures or catheter exchanges.

#### 3.4.3. Biliary Drainage in Acute Cholangitis

High-risk patients should be identified early to allow ICU admission and administer emergency bile duct drainage. Endoscopic techniques are the procedure of choice for drainage of the biliary tree in acute cholangitis, as surgical or percutaneous decompression has higher morbidity and mortality [65]. In critically ill patients, it may be advisable to first place a nasobiliary drain or biliary stent until the patient is stabilized [66]. For definitive treatment of the cause of obstruction, early ERCP with sphincterotomy and stone removal should be chosen. In some cases, it is necessary to insert a biliary stent to ensure proper biliary drainage. Today, emergency surgery is only indicated when endoscopic or percutaneous techniques are unavailable since, in patients with risk factors, the operative morbidity and mortality rates are 91% and 55%, respectively [67].

**Figure 3:**
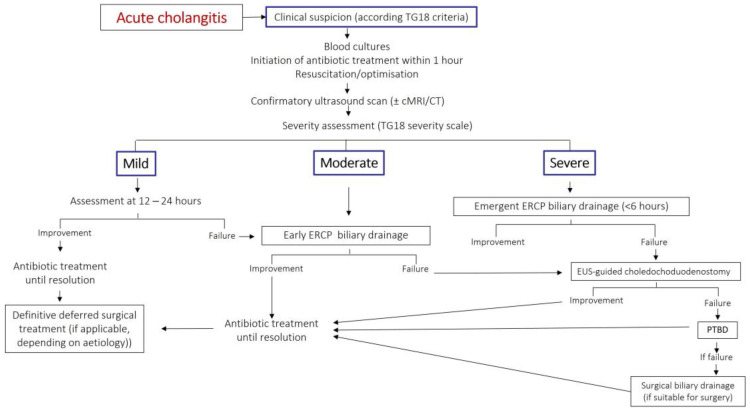
Authors’ proposal for a treatment algorithm for acute cholangitis. Modified from Okamoto K et al. [55]. TG18: Tokyo guidelines 2018. cMRI: cholangio-Magnetic Resonance Imaging. CT: computerized tomography. ERCP: endoscopic retrograde cholangio-pancreatography. EUS: endoscopic ultrasound. PTBD: percutaneous bile duct drainage. ASA: American Society of Anesthesiologists surgical risk classification.

Biliary drainage should be performed within six hours in patients with hemodynamic instability or septic shock [68]. A meta-analysis found a 20% reduction in mortality when ERCP is performed <24 h compared to ≥24 h [69]. Figure 3 reflects a proposed management algorithm for acute cholangitis.

For decades, the second-line therapeutic intervention for biliary drainage after failed ERCP has been PTBD, although it may present complications. In recent years, ultrasound-guided endoscopic biliary drainage (EUS-EBD) has been recognized as an alternative to PTBD for relieving obstruction after failed or non-feasible ERCP [70]. This procedure combines the benefits of endoscopy and ultrasound guidance to precisely target and treat complex biliary pathologies. Two main approaches are commonly employed: the EUS-guided rendezvous technique (EUS-RV) and EUS-guided hepaticogastrostomy or choledochoduodenostomy (EUS-HGS/CD). Several meta-analyses have demonstrated the efficacy of EUS-EBD in achieving successful biliary drainage. Success rates for EUS-RV and EUS-HGS/CD have been reported to range from 80% to 95%, depending on patient characteristics and operator expertise [71,72]. The procedure has shown promising results in various clinical scenarios, including palliation of malignant biliary obstruction, treatment of benign biliary strictures, and management of complications following liver transplantation. While EUS-BD offers notable benefits, certain factors must be considered; for example, operator expertise and experience are crucial to achieving optimal outcomes, as the technique requires advanced endoscopic skills and knowledge of the biliary anatomy.

### 3.5. Special Situations

#### 3.5.1. Gangrenous Cholecystitis

Gangrenous cholecystitis is an advanced stage of cholecystitis that occurs in 30% of cases and requires urgent surgery. It is most common in men over 50 years of age with cardiovascular disease. Characteristic ultrasound signs include a significant irregularity of the gallbladder wall, with numerous striations or asymmetrical thickening, and the presence of intraluminal membranes. It should be noted that Murphy’s sign may be negative due to gallbladder denervation [73,74].

#### 3.5.2. Emphysematous Cholecystitis

This rare distinct form of acute cholecystitis is defined by the presence of intraluminal air or air in the gallbladder wall due to the microbiological etiology of gas-producing bacteria, usually Clostridium welchii (45%), Escherichia coli (30%), or Clostridium perfringens. It is more frequent in diabetic patients, and in 50% of cases, it is associated with gallbladder stones [75]. Typical imaging findings are a balloon-like air picture in the gallbladder area, submucosal or intramural gas, or pericholecystic air [76]. The risk of gallbladder perforation in emphysematous cholecystitis is up to five times that recorded in ordinary cholecystitis, and mortality is high. Emergency surgery should be performed, and treatment against Gram-negative and anaerobic bacteria should be initiated. A combination of a 3rd or 4th generation cephalosporin with metronidazole or monotherapy with ertapenem or piperacillin-tazobactam may be preferred [77].

#### 3.5.3. Acute Acalculous Cholecystitis

Acute acalculous cholecystitis (AAC) has increased in frequency in recent decades. It is a condition with high mortality, high incidence of gallbladder gangrene (50%), and perforation (10–15%). It can be seen in two forms: primary AAC added to an already present severe disease and acute cholecystitis secondary to systemic infection. Cases have been reported as postoperative complications of heart transplants, conventional cardiac surgeries, aortic aneurysm surgeries, traumas, and severe burns. Secondary AAC may complicate systemic bacterial, fungal, or viral infections, *Salmonella typhi*, brucellosis, disseminated candidiasis, systemic leptospirosis and cytomegalovirus (CMV), hepatitis, or Epstein–Barr virus infections [78].

It has been suggested that ischemia of the gallbladder wall plays a major role in the pathogenesis of AAC, which would explain the frequency of mucosal necrosis, arteriolar thrombosis, gallbladder gangrene, and perforation with biliary peritonitis. Other interacting factors may include increased intraluminal pressure, bile stasis secondary to fasting and gastrointestinal hypomotility, cystic duct compression, and spasm of the sphincter of Oddi secondary to opioid analgesics. Similar to calculous cholecystitis, bacterial infection occurs secondarily [79].

In critically ill patients admitted to the ICU with non-specific signs of SIRS or sepsis and who are unable to communicate their complaints, diagnosis is challenging [80]. Ultrasound, which can be performed at the bedside, is the most appropriate diagnostic test, with sensitivity and specificity both above 80%. Ultrasound diagnosis combines findings of hydrops, thickened bile or biliary sludge, and increased gallbladder wall thickness (>3.5 mm), although many patients admitted to the ICU may present with gallbladder wall edema due to causes other than AAC. A CT scan can assess pericholecystic inflammation and even show wall changes, fluid collections, or perforations not seen by ultrasound.

The method of choice for source control is cholecystectomy, although, in patients with hemodynamic instability or organ failure, a percutaneous cholecystostomy will be effective in 85% of cases [58,79]. When cholecystostomy is performed, a rapid improvement of symptoms is expected; otherwise, surgery should be performed without delay. Laparoscopic cholecystostomy, which in extreme cases can be performed at the bedside in the ICU, also allows evaluation of the rest of the abdomen. The main limitation of cholecystostomy is the existence of gangrenous or perforated cholecystitis, which necessitates cholecystectomy [81].

**Table 5:** Summary of suggested empirical antibiotic treatment of biliary infection. Modified from Amillo-Zaragüeta et al. [82].

ORIGIN	Community-Acquired		Health Care-Associated Infections
DIAGNOSE	Acute Calculous Cholecystitis	Acute Calculous Cholecystitis Acute Cholangitis (c)	Acalculous Cholecystitis in Critical Patient Cholangitis with Biliary Stent Cholangitis after ERCP or PTHC
SEVERITY	MILD-MODERATE	SEVERE	
WITHOUT Risk factors of poor evolution (a)	Amoxicillin-clavulanate± gentamicin (b) or Ertapenem or Cephalosporin 2^nd^ + metronidazole Gentamicin or aztreonam + metronidazole *	Piperacilin-tazobactam or Meropenem, imipenem or doripenem (d) Tigecycline * ± Aztreonam or Amikacin (d)	Piperacilin-tazobactam ± amikacin (d) or Meropenem, imipenem or doripenem (d) ± Linezolid, daptomicine, or glycopeptide ± Fluconazole or candin (e) or Tigecyclin (d) + ceftazidime or amikacin o colistine ± Fluconazole or candin (e) *Tigecyclin + Amikacin* *± Fluconazole or echinocandin* (e) *
WITH Risk factors of poor evolution (a)	Ertapenem Tigecycline *	Meropenem or imipenem(d) or Tigecycline + ceftazidime, cefepime or amikacin Tigecyclin + Aztreonam or Amikacin *	

(a) According to the criteria in Table 4. (b) In some areas, 15–25% of *E. coli* strains are resistant to amoxicillin-clavulanate, and an aminoglycoside should be associated. (c) Acute cholangitis is considered a potentially serious infection, requiring coverage of enterococci and anaerobes. (d) In patients at risk of infection by *Enterobacteria* resistant to cefotaxime or *P. aeruginosa* (nosocomial infection with previous antibiotic treatment, neutropenia, history of ERCP/biliary tract drainage) or those presenting septic shock, initial therapy should be considered with a carbapenem or a specific anti-*Pseudomonas* drug such as amikacin, ceftazidime or cefepime. The administration of colistin should be considered in patients previously treated with an antibiotic with anti-*Pseudomonas* activity and who present persistence or recurrence of IIA. (e) In patients at risk of biliary infection involving *Candida* spp. (acalculous cholecystitis in the critically ill patient) an antifungal (fluconazole or echinocandin) should be added to the treatment. Echinocandin is indicated in patients with severe sepsis or septic shock and in those who have previously received fluconazole. * In italics: alternative pattern in betalactam allergy. The ± sign indicates the possibility of additional treatment. ERCP: Endoscopic retrograde cholangio-pancreatography. PTHC: Percutaneous transhepatic cholangiography.

## 4. Conclusions

Severe biliary tract infection is an increasingly common cause of sepsis and carries a high rate of morbidity and mortality. The implementation of international guidelines that categorize its severity according to clinical presentation, laboratory tests, and imaging should allow its timely diagnosis and treatment. However, the implementation of these guidelines is neither widespread nor homogeneous. Sepsis or septic shock requires prioritizing accurate diagnosis, resuscitation, initiation of empirical antimicrobial treatment, and source control. Risk factors for the presence of uncommon organisms, such as ESBL-producing enterobacteriaceae, *Enterococcus faecium,* or *Pseudomonas* spp., should be considered before initiating empirical antibiotics. Early laparoscopic cholecystectomy is the standard treatment for calculous cholecystitis, while early endoscopic drainage techniques are preferred for acute cholangitis. An image-guided percutaneous cholecystostomy is a valuable option for calculous or acalculous cholecystitis in patients at high surgical risk. Table 6 summarizes the main novelties in the management of biliary infection over the last decade.

## Figures and Tables

**Table 1:** Diagnostic criteria and severity classification of acute cholecystitis of TG18/TG13. Modified from Yokoe et al. [19].

**DIAGNOSIS** ***Suspicion***: one criterion A + one criterion B ***Definitive diagnosis***: one criterion A + one criterion B + one criterion C	**A. Local inflammation** A-1 Murphy’s sign A-2 Pain/mass/tenderness in upper right quadrant	**B. Systemic inflammation** B-1 Fever B-2 Elevated CPR B-3 Elevated leukocyte count	**C. Image** C-1 Characteristic findings of acute cholecystitis
**SEVERITY**	**SEVERE (grade III)**	**MODERATE (grade II)**	**MILD (grade I)**
	It is associated with dysfunction in one of the following organs/systems 1. Cardiovascular: hypotension requiring dopamine > 5 µg/kg/min, or any dose of norepinephrine 2. Neurological: decrease in the level of consciousness 3. Respiratory: PaO_2_/FiO_2_ ratio <300 4. Renal: oliguria, creatinine > 2.0 mg/dL 5. Liver: PT-INR > 1.5 6. Haematological: platelet count < 100,000 mm^3^	It is associated with one of the following: 1. Leukocytosis (>18,000/mm^3^) 2. Palpable mass with tenderness in upper right quadrant 3. Duration of symptoms > 72 h 4. Marked local inflammation (gangrenous cholecystitis, pericholecystic abscess, liver abscess, biliary peritonitis, emphysematous cholecystitis)	Does not meet criteria for severe or moderate cholecystitis. It can be defined as acute cholecystitis in a healthy patient without organic dysfunction and with mild inflammatory changes in the gallbladder

**Table 2:** Diagnostic criteria and severity classification for acute cholangitis from TG18/TG13. Modified from Kiriyama et al. [4].

**DIAGNOSIS** ***Suspicion***: one criterion A + one criterion B or C ***Definitive diagnosis:*** one criterion A + one criterion B + one criterion C	**A. Systemic inflammation** A-1 Fever and/or chills A-2 Inflammatory response (CPR elevation, elevated leukocyte count)	**B. Cholestasis** B-1 Jaundice (bilirubin ≥ 2 mg/dL) B-2 Altered liver function (elevated alkaline phosphatases, gamma-GT, and/or transaminases)	**C**. **Image** C-1 Bile duct dilation C-2 Evidence of etiology (stricture, lithiasis, stent)
**SEVERITY**	**SEVERE (grade III)**	**MODERATE (grade II)**	**MILD (grade I)**
	***Dysfunction of one of the following organs/systems*:** 1. Cardiovascular dysfunction: hypotension requiring dopamine ≥ 5 µg/kg per min, or any dose of norepinephrine 2. Neurological dysfunction: disturbance of consciousness 3. Respiratory dysfunction: PaO_2_/FiO_2_ ratio < 300 4. Renal dysfunction: oliguria, serum creatinine > 2.0 mg/dL 5. Hepatic dysfunction: PT-INR > 1.5 6. Haematological dysfunction: platelet count < 100,000/mm^3^	***Two of the following conditions*:** 1. abnormal WBC count (>12,000/mm^3^ or <4000/mm^3^) 2. High fever ≥ 39 °C 3. Age ≥ 75 years 4. Hyperbilirubinemia (total bilirubin ≥ 5 mg/dL) 5. Hypoalbuminemia	“Grade I” acute cholangitis does not meet the criteria of “Grade II” or “Grade III” acute cholangitis.

**Table 3:** Microbiology of bile and blood cultures in patients with biliary infections. Modified from Tokyo Guidelines 2018 [44].

Microorganisms	Proportion of Isolates		
	Bile (%)	Blood Culture (%)	
		Community-Acquired	Nosocomial
Gram-negative			
* E* *scherichia coli*	31–44	35–62	23
* Klebsiella* spp.	9–20	12–28	16
* Pseudomonas* spp.	0.5–19	4–14	17
* Enterobacter* spp.	5–9	2–7	7
* Acinetobacter* spp.	-	3	7
* Citrobacter* spp.	-	2–6	5
Gram-positive			
* Enterococcus* spp.	3–34	10–23	20
* Streptococcus* spp.	2–10	6–9	5
* Staphylococcus* spp.	0	2	4
Anaerobes	4–20	1	2
Others	-	17	11

**Table 4:** Risk factors for poor evolution in biliary infection.

Related to the inadequacy of antibiotic treatment	Risk of infection by unusual organisms (Enterobacteria-ESBL, *Pseudomonas* spp.)
	Hospitalisation > 5 days
	Antibiotic treatment > 3–5 days in the last 6 weeks
	Biliary stent
	Cholangitis after ERCP ^1^
Related to the severity of infection	Sepsis, septic shock
Related with comorbidities	Immunosuppression Malnutrition Diabetes Chronic renal failure Chronic obstructive pulmonary disease Liver cirrhosis
Age-related	>70 years old

^1^ ERCP: Endoscopic retrograde cholangio-pancreatography.

**Table 6:** Main novelties in the management of biliary infection over the last decade. Modified from Amillo-Zaragüeta et al. [82].

Increasing isolation of *Enterococcus* spp., *Enterococcus faecium,* and *Pseudomonas* in acute cholangitis.
Increased resistance of *E. coli* and *Klebsiella* spp. to quinolones.
QuickSOFA (qSOFA) proposed as a new bedside score to identify sepsis (2016) but not recommended as an alternative to SIRS, NEWS, MEWS by the Surviving Sepsis Campaign 2021.
Surviving Sepsis Campaign “Hour-1 bundle” (revised 2021): Measure lactate levels; Blood cultures prior to antibiotic administration; Administer broad-spectrum antibiotics; Rapidly administer 30 mL/kg crystalloids if hypotension or lactate ≥ 4 mmol/L; Administer vasopressors if patient is hypotensive during or after fluid resuscitation to maintain MAP ≥ 65 mm.
Changes in Tokyo Guidelines 2007, 2013, and 2018 (TG07, TG13, TG18) The diagnostic criteria for acute cholecystitis in TG13 have better specificity and higher diagnostic accuracy than the first edition (TG07). The severity classification of acute cholecystitis in TG13 and TG18 is recommended unchanged. The diagnostic criteria for acute cholangitis TG13 are recommended as TG18 criteria. The TG13 acute cholangitis severity classification criteria are recommended as TG18 criteria and may be useful for indicating biliary drainage in grade II patients. MRI/CPMRI recommended for the diagnosis of acute cholangitis in TG18, as they are useful for diagnosing the cause and for assessing inflammation.
Role of endoscopic ultrasound (EUS) in the diagnosis of choledocholithiasis.
Early laparoscopic cholecystectomy is recommended in acute cholecystitis.
Cholecystostomy is recommended in unstable patients with acute cholecystitis.
Early biliary drainage is recommended in acute cholangitis.
New endoscopic techniques combined with EUS are incorporated for drainage of the gallbladder or bile ducts.

SIRS: Systemic Inflammatory Response Syndrome. NEWS: National Early Warning Score. MEWS: Modified Early Warning Score. MRI: Magnetic Resonance Imaging. CPMRI: Magnetic Resonance cholangiopancreatography.

## Data Availability

No new data were created or analyzed in this study. Data sharing is not applicable to this article.
